# Validation of Dietary Vitamin D Intake from Two Food Frequency Questionnaires, Using Food Records and the Biomarker 25-Hydroxyvitamin D among Pregnant Women

**DOI:** 10.3390/nu10060745

**Published:** 2018-06-08

**Authors:** Linnea Bärebring, Anna Amberntsson, Anna Winkvist, Hanna Augustin

**Affiliations:** Department of Internal Medicine and Clinical Nutrition, Sahlgrenska Academy, University of Gothenburg, 40530 Gothenburg, Sweden; linnea.barebring@gu.se (L.B.); anna.amberntsson@hotmail.com (A.A.); anna.winkvist@nutrition.gu.se (A.W.)

**Keywords:** Vitamin D, dietary assessment, biomarker, validation

## Abstract

Our objective was to validate vitamin D intake from a short vitamin D questionnaire (VDQ) and a longer online food frequency questionnaire (FFQ) against a food record and 25-hydroxyvitamin D (25OHD) as a biomarker of vitamin D status, among pregnant women in Sweden. The number of women included was 1125 with VDQ, FFQ and 25OHD, and of those, 64 also completed the food record. Median vitamin D intakes were 3.9 µg by VDQ (*p* < 0.001), and 5.3 µg by FFQ (*p* = 0.89), compared to 5.0 µg by food record. Correlations between vitamin D intake from food record and VDQ (rho = 0.51, *p* < 0.001) or FFQ (rho = 0.49, *p* < 0.001) were similar. The VDQ and FFQ also had a similar ability to rank the individuals according to vitamin D intake. However, only vitamin D intake from the VDQ was significantly associated with vitamin D status as assessed by 25OHD. The validation coefficient for the VDQ was 0.68 and 0.75 for the FFQ. In conclusion, assessing dietary vitamin D intake is challenging, regardless of the dietary assessment method. The VDQ, that includes only four food items, is a valid, simple and useful tool in assessing vitamin D intake of pregnant women in Sweden, while imposing a minimal burden on women and researchers.

## 1. Introduction

Vitamin D intake from food contributes to vitamin D status, together with supplements and sun exposure [1]. At higher latitudes, vitamin D intake is an important source of vitamin D as skin synthesis of vitamin D is not possible year-round, due to insufficient UV-B radiation during winter. In these regions especially, estimation of dietary intake of vitamin D is important. However, no optimal assessment method for use on larger populations exists.

Objective methods to assess dietary intake exist in the form of biomarkers, but only for a few nutrients or foods. Additionally, some of these biomarkers are costly to collect and analyze and require great effort from the participants. Therefore, large scale research studies have a need for valid subjective dietary assessment methods that are easy to use. The food record method, where all food and beverages consumed are recorded in detail during one or several days, is regarded as the optimal subjective dietary intake assessment method because it is based on actual intake and provides information on absolute rather than relative intake [2]. It is however unclear if a food record is the appropriate method for assessment of vitamin D intake, as dietary intake of vitamin D may have a large day-to-day variation, dependent on e.g., fish intake, and the diversity of fortified foods. In addition, the food record method is best suited for nutrients present in a wide range of foods and this is not the case for vitamin D [2]. Further, the method places a considerable burden on the participants and requires resources to handle the data for the researchers if an automated version is not used. Still, the method is considered the gold standard for dietary assessment. In contrast to the food record, the food frequency questionnaire (FFQ) method requires less effort from participants and researchers. Here, participants answer how often they consume predefined foods, thus reflecting intake over a longer period of time. It yields relative intake information and precision on the individual level is lower [2].

Acceptable associations have been reported between vitamin D intake data from FFQs and food records, as well as between FFQ-derived data and the biomarker 25-hydroxyvitamin D (25OHD) as a proxy for vitamin D status. An Irish study found that vitamin D intake derived from a 66 item FFQ correlated well with the intake from a 14-day diet history (*r* = 0.7) and with wintertime 25OHD (*r* = 0.3) [3]. Studies from the US, UK and Canada have found correlation coefficients varying from 0.43 to 0.75 when comparing vitamin D intake from 17–37 item FFQs to food records [4,5,6]. Correlation coefficients were approximately 0.5 when vitamin D intake (including supplements) from FFQs were compared to 25OHD in serum or plasma [4,5,7]. However, one of the studies found no significant correlation between vitamin D intake assessed by a 26 item FFQ and 25OHD [6], and correlations seem to be strongly attenuated among non-supplement users [7]. Hence, further validations of FFQ are needed.

A Swedish national dietary survey, using a 4-day food record, showed that mean vitamin D intake among women of the ages 18–30 years was 5.2 µg (median 4.4 µg) and among women of the ages 31–44 6.2 µg (median 5.1) [8]. Importantly, the largest sources of dietary vitamin D were fish (32%), spread margarine (14%) and dairy products (12%). The fact that vitamin D intake seems to be concentrated to a few food sources suggests that a short FFQ covering only these food items might be a useful and easy tool to assess vitamin D intake in a Swedish setting. We previously found, in a small study, that a vitamin D questionnaire (VDQ) consisting of only four food items was able to capture the majority of vitamin D intake concurrently reported in food records [9]. Hence, a short VDQ seems to be a promising tool for assessing vitamin D intake in populations and deserves further evaluation.

The aim of this paper was to validate vitamin D intake from a VDQ including four foods and a longer online FFQ, with a 4-day food record as a subjective dietary intake reference method, and 25OHD serum concentration as an objective biomarker of vitamin D status among pregnant women in Sweden.

## 2. Materials and Methods

### 2.1. Recruitment and Study Protocol

This study uses data from the Swedish GraviD cohort, which has been previously described [1]. The population-based cohort is unique in that several methods to assess vitamin D intake and vitamin D status were applied. Briefly, pregnant women were recruited at registration to antenatal care. In the third trimester of pregnancy (mean gestational week 33), the women had a blood sample drawn and answered a short questionnaire on vitamin D exposure, including four questions on dietary intake of vitamin D which make up the VDQ. At the same visit, all women were also asked to complete an online full-length FFQ and a subgroup of women were asked to complete a 4-day food record. This study was conducted according to the Declaration of Helsinki and all procedures were approved by the Regional Ethics Committee in Gothenburg. Written and informed consent was provided by all participants.

### 2.2. The VDQ

All women were asked to complete the VDQ during a routine visit to the antenatal care, with assistance by midwives. Interpreters were present when needed, in line with standard care.

In the VDQ, four foods were included: oily fish, milk, yoghurt/sour-milk and margarine. Participants were asked if they ever consumed the food (yes/no) and if they did, additional questions were asked (Supplementary Figure S1). The VDQ took approximately 1–2 min to complete.

Oily fish was assumed to contain 15.63 µg vitamin D per serving (corresponds to 125 g of salmon). Full fat milk was assumed to contain 0.02 µg/100 g. Medium fat, low fat and skim milk were assumed to contain 0.5 µg/100 g. Plant-based milk replacement products were assumed to contain 1.5 µg/100 g unless it was stated that the product was organic, in which case it was assumed not to contain vitamin D as these products are not fortified. Yoghurt/sour milk was assumed to contain 0.38 µg vitamin D/100 g if plain/neutral. Flavored products and Greek/Turkish yoghurt (ca. 10% fat) were assumed not to contain vitamin D. Margarine was assumed to contain 10 µg vitamin D/100 g. One serving of milk or yoghurt/sour-milk was 500, 200 or 50 g depending on the answer. One serving of margarine as spread was 8 g. The maximum vitamin D intake using these assumptions would be 14.95 µg per day. A total of 1801 women completed the VDQ.

### 2.3. The FFQ

The women were given written information about the FFQ (called MealQ) and how it could be accessed online.

The FFQ was an interactive 174-item FFQ, available in Swedish [10] and took approximately 20–30 min to complete. The FFQ has previously been validated with respect to vitamin D intake against food records in healthy volunteers (most with a background in nutrition), with a correlation coefficient of 0.3 [11]. The FFQ was accessed by 1238 women, and completed by 1160.

### 2.4. Food Record

The 4-day food record was in pen-paper format with instructions and a return envelope in which to submit the finished record.

All women from five of the antenatal clinics (*N* = 428) were asked to complete a 4-day food record, in addition to the VDQ and FFQ. The five clinics were chosen to represent women from the whole cohort, on the basis of ethnicity and education level. The women were instructed to describe all food and drink consumed in as much detail as possible. Amounts were weighed if they had access to a scale, or estimated using household measures or provided illustrations of different portion sizes. A total of 85 women provided a food record, 84 of which provided a complete 4-day food record. Vitamin D intake from the food records was calculated by a dietitian, using the program Dietist XP version 3.2 (Kost och Näringsdata AB, Stockholm, Sweden), based on the Swedish National Food Agency’s database (version 2013-10-04).

### 2.5. Blood Sampling and Laboratory Analysis

Venous non-fasting blood samples were drawn at the antenatal care in gestational week 32–40 when the VDQ was administered. Blood was centrifuged for 10 min within 0.5–2 h of sampling. Serum was extracted and stored at −70 °C until analysis by liquid chromatography tandem mass spectrometry at the central laboratory in Malmö. This is the preferred method in the absence of a gold standard. Values are given as the sum of 25OHD_3_ and 25OHD_2_ [12]. The method has previously been described in detail [1].

### 2.6. Statistics

The vitamin D intakes from the VDQ and FFQ were compared to the vitamin D intake from the food record, using related samples Wilcoxon signed ranks test and correlation analysis. In the comparison of subjective methods vs. the objective biomarker, intakes were correlated with 25OHD. Subgroup analyses were also performed on women sampled during winter and among non-supplement users. The validity coefficient for the dietary assessment methods was calculated using the methods of triads, in the whole group and in the subgroup sampled in winter and among non-supplement users [4]. In the calculations of validity coefficients, only participants with complete data were included in the correlations. Lastly, intakes of vitamin D from each method and 25OHD concentrations were ranked and divided into quartiles, to compare the ability to rank individuals according to vitamin D intake or vitamin D status.

## 3. Results

A total of 1125 women completed both the VDQ, the FFQ and had measures of 25OHD, and were thus included in this study. A subgroup of 64 women had also completed the food record. The characteristics of the women are shown in Table 1.

### 3.1. Food Record as Reference

Median (interquartile range) dietary vitamin D intake among the 64 women was 5.0 (3.6–9.0) assessed by food record, 5.3 (3.8–7.0) assessed by FFQ and 3.9 (2.8–5.4) assessed by VDQ. The vitamin D intake from the food record differed significantly from the VDQ (*p* < 0.001) but not from the FFQ (*p* = 0.89). The largest contributor to vitamin D intake was oily fish (28%), followed by margarine (16%), milk (10%), lean fish (8%) and yoghurt/sour-milk (2%). Thus, the four foods included in the VDQ (oily fish, milk, margarine and yoghurt/sour-milk) made up 56% of total vitamin D intake in the food records.

The vitamin D intake from the food record correlated with intake from both the VDQ (rho = 0.51, *p* < 0.001) and the FFQ (rho = 0.49, *p* < 0.001) (Table 2). In comparisons of the methods’ abilities to rank individuals according to vitamin D intake, the food record and the VDQ ranked 39% of the participants in the same quartile, 42% in the adjacent quartile, 14% two quartiles apart and 5% in the opposite quartile. When comparing the FFQ and the food record, 41% were in the same quartile, 44% in the adjacent quartile, 12% two quartiles apart and 3% in the opposite quartile.

The vitamin D intake from the VDQ and FFQ correlated significantly (rho = 0.235, *p* < 0.001) and the median intake was significantly lower when assessed by VDQ (*p* < 0.001). When comparing the two methods ability to rank the individuals by quartiles of vitamin D intake, 44% were placed in the same quartile, 31% in the adjacent quartile, 22% two quartiles apart, and 3% in the opposite quartile.

### 3.2. Biomarker 25-Hydroxyvitamin D as Reference

Among the 1125 women, concentrations of 25OHD correlated significantly with vitamin D intake assessed by VDQ, but not by FFQ (Table 2). This was also true during wintertime and/or among non-supplement users. Additionally, vitamin D intake from the food record (*N* = 64) was not significantly related to 25OHD overall or 25OHD during winter, but was significant among non-supplement users.

When ranking vitamin D intake from all three methods and comparing it to the ranking of vitamin D status, the VDQ placed 29% in the same quartile, 37% in the adjacent quartile and 8% in the opposite quartile (Table 3). The FFQ placed 25% in the same quartile, 37% in the adjacent quartile and 8% in the opposite quartile, compared to 25OHD concentration. Finally, the food record placed 27% in the same quartile, 40% in the adjacent quartile and 11% in the opposite quartile.

The validity coefficient for the VDQ was 0.68 overall, and 0.60 among wintertime 25OHD samples. Validity coefficients for the FFQ was 0.75 overall, and 0.60 during wintertime. For the food record, the validity coefficient was 0.66 overall and 0.80 during wintertime. Among non-supplement users the validity coefficients were 0.57 for the VDQ, 0.57 for the FFQ and 0.85 for the food record.

## 4. Discussion

We found that, in Swedish pregnant women, vitamin D intake reported by the VDQ correlated well with the intake from a food record as well as with vitamin D status as reflected in circulating 25OHD concentrations. As expected, median vitamin D intake was lower when assessed by VDQ than by food records. Estimated vitamin D intake by the longer FFQ was not reflected in vitamin D status which could indicate that the longer FFQ might not be ideal to capture habitual vitamin D intake. 

The VDQ does not include all dietary sources of vitamin D, which is evident by the lower median intake compared to the results from the food record. Despite the fact that the majority of dietary vitamin D is included in the VDQ, the method underestimates total dietary vitamin D intake. On the other hand, an assessment of vitamin D intake from all dietary sources is usually based on assumptions that may very well overestimate vitamin D intake. By limiting the number of foods included in the VDQ to the most essential sources, this problem is minimized. For instance, baked goods or cooking fat can contain significant amounts of vitamin D, which indicates that fortified margarine and not butter is assumed to be used. These assumptions and similar ones are likely to add up and have the potential to cause a significant overestimation of vitamin D intake. The VDQ is likely to be less sensitive to these assessment errors, as no such assumptions are made. A disadvantage in using the VDQ is that the tool does not provide an estimate of energy intake, and the vitamin D intake can therefore not be energy adjusted. Moreover, the method does not provide estimates of other nutrients other than vitamin D.

The correlations between vitamin D intake from both the VDQ and FFQ with the food record were in line with previous research reporting correlations between 0.43–0.75 [4,5,6,7,13]. The association between vitamin D status and vitamin D intake seems largely dependent on the dietary assessment methodology. Overall vitamin D intake and 25OHD concentration were only significantly related when vitamin D intake was assessed by VDQ. However, the association was quite weak and the proportion of variation in 25OHD explained by vitamin D intake was small. This is most likely explained by the fact that sun exposure and supplement use are larger contributors to vitamin D status than dietary intake. Indeed, correlation between 25OHD and vitamin D intake among non-supplement users was significant by food record and trending toward significance also for the FFQ. A Finnish study found a correlation of 0.28 between vitamin D intake from a 98-item FFQ and 25OHD among non-supplement users during wintertime [7]. This is comparable to our findings for the VDQ. The vitamin D intake from the FFQ was not associated with vitamin D status overall or during winter. The validity coefficients for the VDQ and FFQ were high at 0.68 and 0.75 overall, which is comparable to previous studies using more extensive FFQs [3,4,7]. The ability to rank the participants according to quartiles of intake was comparable between the VDQ and FFQ, using either vitamin D status and food record as reference.

The overall vitamin D intake estimated from the food record was in line with the national survey by the National Food Agency [8]. However, fish intake seemed to contribute a bit more to total vitamin D intake in the GraviD cohort which might indicate that pregnant women alter their diet slightly, or that the women in the study over-reported their fish intake. This is not implausible, since they were informed that the purpose was to measure their vitamin D intake and status. It is also possible that pregnant women have higher intakes of fish than non-pregnant women.

A limitation in this study is that only a subset of 64 women completed the food record. Still, statistical power seems sufficient for our analyses, as significant differences and significant correlations between the methods could be seen. These 64 women also seem comparable to the overall cohort in terms of age, BMI, 25OHD concentration, tobacco use, education and ethnicity [1]. Data on vitamin D status were available from all 1125 women who completed the VDQ and FFQ. These women also seem comparable to the overall cohort, apart from a higher proportion born in Northern Europe, 86% compared to 75% in the whole cohort [1]. Even though the women included in this paper seem to represent the women in the original cohort and thus pregnant women overall in Sweden, our results need verification in other groups to ensure generalizability of the findings. Lastly, vitamin D status does not only reflect vitamin D intake from food but also supplement use and sun exposure. Therefore, 25OHD may be a poor biomarker for dietary vitamin D intake. However, as all three dietary assessments were carried out during a time period of a few weeks, this probably did not affect the comparisons of the results, as supplementation and sun exposure likely remained constant. Many studies have previously shown that 25OHD concentrations reflect dietary vitamin D intake [3,4,5,6,7], and has thereby been proven useful as a biomarker of intake.

Strengths of this study are the large number of participants in this population-based cohort and that three different dietary assessments were used, which provides important information. Also, using a biomarker as a reference method provides results that are not subject to recall bias or misreporting. We did not include supplementary intake of vitamin D in the estimate of vitamin D intake. Only dietary sources were included, as these were considered more challenging to capture than supplementary intake, especially among pregnant women who have a frequent and consistent use of supplementary vitamin D throughout pregnancy [14]. Supplementary intake is thus likely easily assessed by a questionnaire or interview, and can be added to dietary intake to estimate total intake in situations where this is desired.

## 5. Conclusions

Estimation of dietary vitamin D intake is challenging, regardless of the dietary assessment method. The VDQ, that includes only four food items, is a valid, simple and useful tool in assessing vitamin D intake of pregnant women in Sweden while imposing a minimal burden on women and researchers.

## Figures and Tables

**Table 1:** Characteristics of all women who participated in the study and those who also provided a food record.

	**All (*N* = 1125) Mean (SD)**	**Subgroup with Food Record (*N* = 64) Mean (SD)**
Age T1 (years)	31.9 (4.6)	31.5 (4.9)
Height T1 (cm)	167.3 (6.2)	166.4 (6.1)
BMI T1 (kg/m^2^)	24.1 (4.0)	24.2 (4.3)
25OHD T3 (nmol/L)	80.9 (33.2)	78.5 (31.1)
	**All % (*N*)**	**Food Record % (*N*)**
BMI ≥ 25 T1	31.1 (350)	29.7 (19)
Tobacco use T1	3.9 (44)	4.7 (3)
Unemployment T1	11.3 (127)	18.8 (12)
Born in North Europe	85.6 (963)	78.1 (50)
University education T1	68.7 (773)	61.0 (39)
Nulliparous	42.5 (478)	57.8 (37)
Travelled < 40° T3	19.6 (220)	17.2 (11)
Nov-April T3	58.0 (653)	59.4 (38)
Vitamin D supplement T3	43.6 (490)	39.1 (25)

T1, First trimester, inclusion; T3, third trimester.

**Table 2:** Correlation between the VDQ and FFQ with food records, and with 25-hydroxyvitamin D (25OHD).

		VDQ		FFQ		Food Record	
	*N*	Rho	*p*	Rho	*p*	Rho	*p*
Food record	64	0.51	<0.001	0.49	<0.001	-	-
25OHD	1125	0.137	<0.001	0.036	0.231	0.142 ^1^	0.396
Wintertime 25OHD	653	0.175	<0.001	0.060	0.126	0.102 ^2^	0.426
Non-supplement user 25OHD	635	0.147	<0.001	0.076	0.057	0.417 ^2^	0.009
Non-supplement user wintertime 25OHD	346	0.212	<0.001	0.028	0.610	0.200 ^3^	0.413

^1^*N* = 64; ^2^ *N* = 38; ^3^ *N* = 19.

**Table 3:** Agreement in classification by quartiles, between the three methods of vitamin D intake assessment compared to the biomarker 25-hydroxyvitamin D (25OHD).

	VDQ	Food Record	FFQ
Same quartile as 25OHD	29%	27%	25%
Adjacent quartile as 25OHD	37%	40%	37%
Two quartiles from the same quartile as 25OHD	27%	22%	30%
Opposite quartile as 25OHD	8%	11%	8%

VDQ, Vitamin D questionnaire; FFQ, food frequency questionnaire.

## Supplementary Materials

The following are available online at http://www.mdpi.com/2072-6643/10/6/745/s1, Figure S1: The short vitamin D questionnaire (VDQ).
