# Supplementary material for: Validation of Dietary Vitamin D Intake from Two Food Frequency Questionnaires, Using Food Records and the Biomarker 25-Hydroxyvitamin D among Pregnant Women
Source: Nutrients. 2018 Jun 8;10(6):745. doi: 10.3390/nu10060745 (PMC6024652; doi:10.3390/nu10060745)
Supplement: Supplementary file 1 [file nutrients-10-00745-s001.pdf]

Do you eat **fatty** fish (salmon, mackerel, herring)? ☐NO ☐YES

If YES: How often?

- ☐At least twice weekly
- ☐3-4 times per month
- ☐1-2 times per month
- ☐More seldom

Do you drink milk or is milk part of your diet? ☐NO ☐YES

If YES: How much milk do you consume?

- ☐More than 3 dl (10 oz) per day
- ☐1-3 dl (3.5-10 oz) per day
- ☐Less than 1 dl (3.5 oz) per day

If YES: Which type of milk do you consume most often?

- ☐Full-fat milk (standardmjölk)
- ☐Medium-fat milk (mellanmjölk)
- ☐Low-fat milk (lättmjölk)
- ☐Skim milk (minimjölk)
- ☐Other (e.g. oat milk), please specify:………………………..

Is yoghurt and/or sour milk part of your diet? ☐NO ☐ YES

If YES How much yoghurt/sour milk do you consume?

- ☐More than 3 dl (10 oz) per day
- ☐1-3 dl (3.5-10 oz) per day
- ☐Less than 1 dl (3.5 oz) per day

If YES What kind do you consume most often?

- ☐fruit-/vanilla yoghurt/sour milk
- ☐plain yoghurt/sour milk
- ☐turkish/greek yoghurt

Do you use margarine as a spread (t ex. Bregott, Lätta, Flora, Milda, Becel)? ☐ NO ☐YES

If YES: How many sandwiches with margarine do you eat?

- ☐at least 4/day
- ☐2-3/day
- ☐4-7/week
- ☐1-3/week
- ☐fewer

Supplementary Figure 1. The short vitamin D questionnaire (VDQ).
